# Cellulose Acetate–Ionic Liquid Blends as Potential Polymers for Efficient CO_2_ Separation Membranes

**DOI:** 10.3390/polym16040554

**Published:** 2024-02-18

**Authors:** Giannis Kontos, Costas Tsioptsias, Ioannis Tsivintzelis

**Affiliations:** 1 Department of Chemical Engineering, Aristotle University of Thessaloniki, 54124 Thessaloniki, Greece; iokontos@cheng.auth.gr; 2 Department of Chemical Engineering, University of Western Macedonia, 50132 Kozani, Greece

**Keywords:** membranes for CO_2_ separation, CO_2_ capture, choline glycine synthesis, green ionic liquids, amino acid ionic liquids, immidazolium-based ionic liquid, cellulose acetate, mass loss analysis

## Abstract

CO_2_ capture, applied in CO_2_ separation from natural gas or in CO_2_/N_2_ separation from power plant flue gas streams, is of great importance for technical, economic, and environmental reasons. The latter seems important because CO_2_, as a greenhouse gas, is considered the main contributor to global warming. Using polymeric membranes for CO_2_ separation presents several advantages, such as low energy demand, small equipment volume, and the absence of liquid waste. In this study, two ionic liquids (ILs) were used for the preparation of cellulose acetate (CA)–IL blend membranes for potential CO_2_ capture applications, namely, 1-butyl-3-methylimidazolium hydrogen sulfate ($\left[{\mathrm{Bmim}}^{+}\right]$[${H\mathrm{SO}}_{4}^{-}$]) and choline glycine ($[{\mathrm{Ch}}^{+}]\left[{\mathrm{Gly}}^{-}\right]$), as they present adequate CO_2_ dissolution ability. The first IL is commercially available, whereas the latter was synthesized by a novel route. Several composite membranes were prepared through the solvent casting technique and characterized by a variety of methods, including thermogravimetry, calorimetry, FTIR spectroscopy, and X-ray diffraction. The CO_2_ sorption in the composite membranes was experimentally measured using the mass loss analysis (MLA) technique. The results showed that the ILs strongly interacted with the C=O groups of CA, which exhibited high affinity with CO_2_. In the case of $\left[{\mathrm{Bmim}}^{+}\right]$[${H\mathrm{SO}}_{4}^{-}$], a reduction in the available sites that allow strong intermolecular interactions with CO_2_ resulted in a decrease in CO_2_ sorption compared to that of pure CA. In the case of $[{\mathrm{Ch}}^{+}]\left[{\mathrm{Gly}}^{-}\right]$, the reduction was balanced out by the presence of specific groups in the IL, which presented high affinity with CO_2_. Thus, the CA-$[{\mathrm{Ch}}^{+}]\left[{\mathrm{Gly}}^{-}\right]$ blend membranes exhibited increased CO_2_ sorption capability, in addition to other advantages such as non-toxicity and low cost.

## 1. Introduction

CO_2_ capture is of great importance for technical, economic, and environmental reasons. Natural gas treatment, which includes CO_2_ separation from natural gas, is essential for meeting the pipeline flow specifications and increasing the heating value of the gas [1]. Other examples of industrial processes in which CO_2_ recovery is a crucial part of the overall procedure are CO_2_/N_2_ separation from power plant flue gas streams, CO_2_/H_2_ separation from fuel gas (syngas) in hydrogen production, and CO_2_/O_2_ separation in food packaging [2,3]. On the other hand, CO_2_, as a greenhouse gas, is considered the main contributor to climate change [4]. Different carbon capture (CC) technologies have been developed for CO_2_ recovery from the aforementioned gas streams, such as physical or chemical adsorption by liquid solvents, pressure or temperature swing adsorption, cryogenic distillation, and membrane separation [2,3]. Among the available carbon capture technologies, the absorption−desorption process using alkanolamines is considered the most mature one due to its extensive application for acid gas removal from natural gas streams [1,4]. Also, pressure swing adsorption (PSA) and cryogenic distillation technologies are used for CO_2_/H_2_ separation [5,6]. However, the major drawback of these technologies is their high energy consumption [7]. Also, chemical solvents, such as alkanolamines, due to their high volatility and significant degradation during thermal processes, are considered a potential threat to the environment and human health [8].

Using polymeric membranes for CO_2_ capture is an emerging, environmentally benign separation method due to its inherent merits over traditional chemical absorption, such as low energy demand, easy maintenance, and compactness of the separation medium [9]. Polymeric membranes were first commercialized in the 1980s for CO_2_ removal from natural gas [10]. Among the polymers used in membranes for gas separations are cellulose acetate, polyethersulfone, polyphenyl oxide, polyimides such as Matrimid^®^, and polycarbonates such as Kevlar^®^ [11,12,13] (Figure S1 of the Supplementary Information file). Although such membranes have been used in gas separation processes for decades, their widespread use is limited, mainly due to their low separation efficiency and poor stability [11]. The semi-crystalline nature of these polymers results in decreased CO_2_ solubility, diffusivity, and permeability, increasing the required membrane area and consequently the capital and operational costs [14,15]. Another major drawback is the plasticization of such membranes in the presence of CO_2_ as well as other highly plasticizing components, such as hexane and toluene, included in some gas streams [16,17]. Research efforts have been directed to enhance separation efficiency in terms of the optimal combination of permeability, selectivity, and membrane stability and to reduce the plasticization effect of CO_2_ using various approaches, such as thermal treatment [16], chemical cross-linking [18,19], synthesis of poly-ionic liquids [20], and the use of polymer blends [21,22]. Although these approaches, in general, result in better stability, they are often related to lower gas permeability [11].

The introduction of a liquid into a supporting polymeric membrane (SLM) is a promising approach to improving the efficiency of such membranes because it induces CO_2_ diffusivity and may result in a significant improvement in CO_2_ permeability [23]. Despite such advantages, employing SLMs for CO_2_ capture in large-scale industrial applications is hindered by problems related to long term stability, mainly due to the extraction of the liquid from the polymer matrix [24,25] and solvent losses due to vaporization [26,27]. These drawbacks, combined with solvent losses due to swelling and leaching of the liquid solvent into a contacting liquid phase, for instance, in pervaporation, result in deterioration of the separation performance [24,25]. Also, solvents used in these processes are, in general, volatile, toxic, and highly flammable, thus raising environmental and health concerns [28,29,30,31].

One promising alternative to overcome these drawbacks is using ionic liquids (ILs) as transport media in specially designed polymer membranes (supported IL membranes, SILM) to selectively remove CO_2_ from gas streams. Due to their unique nature, i.e., organic salts consist of ions with a rather high molecular weight, ILs show several interesting properties, including very low vapor pressure [32], non-flammability [33], and high stability at temperatures above 200 ℃ [34]. Along with these properties, their relatively high viscosity results in higher capillary forces with the supporting media [35]. Three main techniques have been reported in the literature for the preparation of supported ionic liquid membranes (SILMs), such as penetration of IL into membrane pores by direct immersion of the polymer into the IL [36], application of vacuum [37], or pressure [38]. Despite their performance, which has often been encouraging [39], SILMs present a major drawback regarding their stability. In addition, the dissolution of the supported liquid phase if such membranes encounter another liquid phase, for instance, in pervaporation, leads to ultimate membrane failure [40].

Another approach to avoiding these stability problems regarding SILMs is the preparation of polymeric room-temperature ionic liquids (PILs) [3]. Although such membranes are more stable with good separation characteristics [3], in some cases, they use particular polymerizable monomers and require complex polymerization techniques; thus, the preparation cost becomes significantly high. The solution casting method used in this work, in which the polymeric matrix is mixed with the ionic liquid and then cast to form a blend film (polymer inclusion membranes, PIMs), is the most cost-effective technique because it is an easy-to-maintain and low-cost route to prepare stable ionic liquid membranes [41].

The selection of the supporting polymer and the liquid medium is the key factor in designing highly efficient membranes for CO_2_ separation. Several polymers, such as polyimides [42], polyvinylidene fluoride [43], and cellulose acetate (CA) [44] were investigated. The latter, CA (Table 1), is a reasonable option for the supporting material due to advantages such as high CO_2_/N_2_ selectivity under common operating conditions and low plasticization induced by heavy hydrocarbons [45]. Also, it is a non-toxic and biodegradable material derived from cellulose, which is an abundant biomaterial, and presents high affinity with many ILs [46]. Such advantages, combined with its low cost, render it a popular polymer matrix for the development of membranes.

Choline (Ch), a nutrient found in many food products [47], is known to be non-toxic and biodegradable [48]. Cholinium-based ILs are biodegradable [49], with low toxicity [48] and high CO_2_ capture efficiency [50]. Thus, the cation of choline can be considered a potential constituent of an efficient IL for CO_2_ removal. Considering the anion of the ionic liquid, CO_2_ measurements in three amino-acid-based ionic liquids with 1-butyl-3-methylimidazolium (Bmim) as cation and glycine (Gly), alanine (Ala), or valine (Val) as anion showed that the glycine-based ionic liquid presented the highest CO_2_ sorption [51]. Furthermore, the number of amine groups in the amino acid anion strongly affects the stoichiometric CO_2_ loading (due to chemical absorption). For example, asparagine-based ILs, with two amine groups in asparagine, follow the 2:1 mechanism (2 mol CO_2_/mol IL) [52], whereas glycine-based ILs, with one amino group in glycine, follow the 1:1 mechanism (1 mol CO_2_/mol IL) [51]. Unlike most ILs, which, in general, show relatively high viscosity [53], choline glycine IL ($[{\mathrm{Ch}}^{+}]\left[{\mathrm{Gly}}^{-}\right]$) (Table 1) presents moderate viscosity [54].

Imidazolium-based ILs show very high SO_2_ solubility, moderate CO_2_ solubility, and relatively poor N_2_ and O_2_ solubilities, suggesting their potential for gas separation [55]. The interaction of CO_2_ (Lewis acid, LA) with the anion of ILs (Lewis base, LB) is an important factor in determining the CO_2_ solubility of ILs. For example, it was shown that there are two contributing factors to the relative high CO_2_ solubility in 1-ethyl-3-methylimidazolium hydrogen sulfate ($\left[{\mathrm{Emim}}^{+}\right]$[${H\mathrm{SO}}_{4}^{-}$]): the high sulfonyl group (S=O) polarization, which leads to stronger intermolecular interactions with CO_2_ [56], and the high negative charge of all oxygen atoms in the HSO_4_^−^ anion, resulting in strong polar interactions with the positively charged carbon atom of CO_2_ [57]. By considering this aspect, the CO_2_ solubility in 1-butyl-3-methylimidazolium hydrogen sulfate ($\left[{\mathrm{Bmim}}^{+}\right]$[${H\mathrm{SO}}_{4}^{-}$]) (Table 1), which has a larger alkyl chain in the cation than the $\left[{\mathrm{Emim}}^{+}\right]$[${H\mathrm{SO}}_{4}^{-}$] and consequently presents a larger free volume, is expected to be higher than the CO_2_ solubility in $\left[{\mathrm{Emim}}^{+}\right]$[${H\mathrm{SO}}_{4}^{-}$] or at least in the same order.

Therefore, in this work, $\left[{\mathrm{Bmim}}^{+}\right]$[${H\mathrm{SO}}_{4}^{-}$] and $[{\mathrm{Ch}}^{+}]\left[{\mathrm{Gly}}^{-}\right]$ (Table 1) were chosen as additives to CA membranes. The aim of this work was to prepare and characterize CA-IL membranes and explore their potential for CO_2_ capture applications.

## 2. Materials and Methods

### 2.1. Materials and Instruments

Potassium bromide (KBr), purity > 99.5% wt., was purchased from Chem-Lab (Zedelgem, Belgium). Cellulose acetate (39.7% wt. acetyl content, with a degree of substitution (*DS*) of 2.45 and *M_n_* equal to 50,000 g mol^−1^) was purchased from Sigma-Aldrich (St. Louis, MO, USA). 1-Butyl-3-methylimidazolium hydrogen sulfate ($\left[{\mathrm{Bmim}}^{+}\right]$[${H\mathrm{SO}}_{4}^{-}$]), purity > 94.5% wt., was purchased from Aldrich. To evaluate the structural characteristics of the CA-IL films, X-ray diffraction (XRD) measurements were carried out at room temperature. The XRD data of sample films (20 mm × 15 mm) were collected by a Brucker (model D8 Advance, Billerica, MA, USA) diffractometer equipped with a Siemens X-ray tube (Cu, 1.54 Å) at a scan range of 5–40° and a rate of 0.5° min^−1^.

FTIR spectrometry using the KBr method was carried out with a Biorad FTS-175 spectrometer (Bio Rad, Hercules, CA, USA). Pieces of the samples were mixed with KBr (mass proportion ~1:200) and pressed into pellets by a hydraulic press (100 Bar). The spectra resulted from an average of 64 scans at 2 cm^−1^ resolution between 400 and 4000 cm^−1^.

Thermogravimetric analysis (TGA) was performed on a thermogravimetry thermal analyzer (Shimadzu TGA-50, Shimadzu, Tokyo, Japan) under a nitrogen gas flow of 20 mL min^−1^. The measurements were conducted with the CA-IL samples heated to 450 °C at a heating rate of 10 °C min^−1^. TGA curves were used to determine the degradation onset temperature using the tangent method (Section S5 in ESI).

To determine the thermal transitions of the CA-IL blends, differential scanning calorimetry (DSC) measurements were carried out by the DSC-50 calorimeter (Shimadzu DSC-50, Shimadzu, Tokyo, Japan) under a nitrogen gas flow of 20 mL min^−1^. The DSC equipment was calibrated using an indium standard (melting point of 156.65 °C, ΔH_f_ = 28.45 J/g), and errors were found to be less than 2% in the heat flow calibration. DSC samples (~1–5 mg) were sealed in an aluminum pan. The measurements were conducted with the samples heated to 250 °C at a heating rate of 10 °C min^−1^.

### 2.2. Synthesis of $[Ch^{+}]\left[Gly^{-}\right]$

$[{\mathrm{Ch}}^{+}]\left[{\mathrm{Gly}}^{-}\right]$ was synthesized using a two-step reaction procedure (Scheme S1 in the Supplementary Information file in Section S2). The first reaction was the metathesis reaction between choline chloride ($[{\mathrm{Ch}}^{+}]\left[{\mathrm{Cl}}^{-}\right]$) and sodium hydroxide (NaOH), which took place in ethanol under stirring for 2 h. Choline hydroxide ($[{\mathrm{Ch}}^{+}]\left[{\mathrm{OH}}^{-}\right]$) remained dissolved, while NaCl precipitated as a white powder (Figure S2). Once NaCl was removed by filtration, glycine (Gly) was added. The second reaction was the neutralization reaction between $[{\mathrm{Ch}}^{+}]\left[{\mathrm{OH}}^{-}\right]$ and glycine to form the desirable $[{\mathrm{Ch}}^{+}]\left[{\mathrm{Gly}}^{-}\right]$ IL and water as a by-product (Figure S2 in the Supplementary Information file). The unreacted amino acid was removed by filtration. The produced water was removed using a rotary evaporator under vacuum for at least 48 h. Three criteria were used for a preliminary identification of the synthesized IL, namely, color, viscosity (evaluated visually), and decomposition temperature, using TGA analysis. The resulting IL was a brown-yellowish, semi-transparent viscous liquid with a decomposition temperature below 180 °C, a value between those of $[{\mathrm{Ch}}^{+}]\left[{\mathrm{OH}}^{-}\right]$ and glycine and close to that reported in the literature for $[{\mathrm{Ch}}^{+}]\left[{\mathrm{Gly}}^{-}\right]$ [58,59]. TGA analysis was also used for an initial estimation of the water content of the produced IL. For an accurate estimation, coulometric Karl Fischer titration was used, and the water content was determined to be less than 1.71% wt. Such a relatively high water content, which remained after an intense purification method, indicates the well-known difficulty in the purification of ILs. The successful $[{\mathrm{Ch}}^{+}]\left[{\mathrm{Gly}}^{-}\right]$ synthesis was finally confirmed by ^1^H and ^13^C NMR analyses. ^1^H and ^13^C NMR data are reported in the Supplementary Information file (Section S2).

### 2.3. CA-IL Film Preparation

The composite CA-$[{\mathrm{Ch}}^{+}]\left[{\mathrm{Gly}}^{-}\right]$ and CA-$\left[{\mathrm{Bmim}}^{+}\right]$[${H\mathrm{SO}}_{4}^{-}$] membranes were prepared through the solvent casting method (Figure S5 in the Supplementary Information file). In more detail, cellulose acetate was dissolved in acetic acid, up to 5% wt., and the ionic liquid was subsequently added, resulting in solutions containing ionic liquid in the range of 0–30% wt. with respect to the polymer weight. The polymer–IL solutions were cast into Petri dishes. After slow drying at ambient temperature for 3 days, the films were further dried in a vacuum at 70 °C for 4 h. Finally, the free-standing films were peeled from the substrate, and they were stored in a vacuum desiccator until use to avoid the sorption of water.

### 2.4. CO_2_ and N_2_ Sorption Measurements and Estimation of Diffusion Coefficients and Permeability

The mass loss analysis (MLA) method [60] was used for measuring the sorption of CO_2_ (or N_2_) by the produced membranes. The experimental apparatus (see Figure S6 in the Supplementary Information file) used to apply the mass loss analysis (MLA) technique consisted of an ISCO high-pressure syringe pump (model 100DX), an ISCO SFX 2-10 thermostatic high-pressure extractor, a high-pressure cell sealed by a screw cap, and a pump/pressure controller. The variable-volume high-pressure cylinder of the syringe pump, filled with liquid CO_2_, was cooled with the aid of a Haake refrigerated circulating bath to keep the temperature constant around −2 °C. After placing a known amount of the polymer sample in the sorption cell, the cell was evacuated to remove any gases that might have been sorbed by the polymer. Then, CO_2_ was introduced and pressurized to the targeted value at a constant temperature of 35 °C. The sample was exposed to CO_2_ until sorption equilibrium was achieved. After preliminary sorption experiments at three different times (30, 60, and 90 min), it was found that 30 min were adequate for the establishment of equilibrium. After equilibrium was reached, the cell was rapidly depressurized, followed by, as quickly as possible, the transfer of the specimen to a fast response electronic balance (precision = 0.0001 g). The time was set equal to zero (*t* = 0) when the valve was opened (start of depressurization). During the desorption of the gas under ambient conditions, the sample weight was recorded as a function of time.

The Fickian diffusion (FD) model assumes that pressure, temperature, and polymer film thickness (L) are constant, and the diffusion is unidirectional. In the case of flat geometry, the CO_2_ uptake at time t ($M_{t})$ and the equilibrium uptake ($M_{\mathrm{eq}}$) are related by the following simplified equation [61]:

(1) $$\frac{M_{t}}{M_{\mathrm{eq}}}=4{\left(\frac{\mathrm{Dt}}{{\pi L}^{2}}\right)}^{1/2}$$

where D is the diffusion coefficient. Equation (1) can be rearranged in terms of the mass of gas remaining in the polymer ($M_{\mathrm{gas}}=M_{\mathrm{eq}}-M_{t}$) as follows:

(2) $$M_{\mathrm{gas}}=M_{\mathrm{eq}}-4{\left(\frac{\mathrm{Dt}}{{\pi L}^{2}}\right)}^{1/2}M_{\mathrm{eq}}$$

This formula allows the determination of the diffusion coefficient D from the slope of a plot of $M_{\mathrm{gas}}$ versus $t^{1/2}$, while $M_{\mathrm{eq}}$ is given by the y-intercept (see Figure S7 in the Supplementary Information file). $M_{\mathrm{eq}}$ was calculated using this approach. For each one of the samples, $M_{\mathrm{eq}}$ was found to depend linearly on pressure, that is, Henry’s law was obeyed. Thus, the solubility coefficient S (Henry’s law constant) can be calculated by the following equation:

(3) $$S=\frac{M_{\mathrm{eq}}}{\mathrm{Pressure}}$$

For the diffusion coefficient, alternatively, if the time at which half of the equilibrium mass has been desorbed is known (that is, the time at which it holds $M_{\mathrm{gas}}=0.5\cdot M_{\mathrm{eq}}$), then D can be calculated by the following equation [62]:

(4) D=0.049tL2for t at which Mgas=0.5· Meq 

In this study, we used Equation (4) for the calculation of the diffusion coefficient D. In each experiment, the thickness (L) of the membrane was measured with a micrometer. In all cases, the thickness was of the same order of magnitude and in the range of 0.1–0.3 mm.

From the value of diffusion coefficient D as well as the value of the solubility coefficient S, the gas permeability P can be calculated from the following equation:

(5) P=D·S

In order to calculate P in its common units, that is, in Barrer ($1\;\mathrm{Barrer}=10^{-10}\frac{{\mathrm{cm}}^{3}\mathrm{STP}\cdot \mathrm{cm}}{{\mathrm{cm}}^{2}\cdot s\cdot \mathrm{cmHg}}$), it is necessary to use cmHg units for Pressure in Equation (3) and to express $M_{\mathrm{eq}}$ in cm^3^ STP/cm^3^. We calculated and presented the results of $M_{\mathrm{eq}}$ in units of g CO_2_/100 g of membrane. In order to transform the g CO_2_/100 g of membrane into cm^3^ STPCO_2_/cm^3^ of membrane, we used a value of 1.977 kg/m^3^ for the STP density of CO_2_ [63] and a value of 1300 kg/m^3^ for the density of cellulose acetate [64]. For the CA-IL membranes, for the sake of simplicity, the same density as that for the pure CA film was used. Because the density of ILs is typically in the range 1000–1400 kg/m^3^ [65], that is, rather close to the density of CA, the above simplification has a very small effect on the values of S.

## 3. Results and Discussion

### 3.1. CA-IL Membrane Characterization

#### 3.1.1. Membrane Structural Properties by X-ray Diffraction Studies

**Figure 1:**
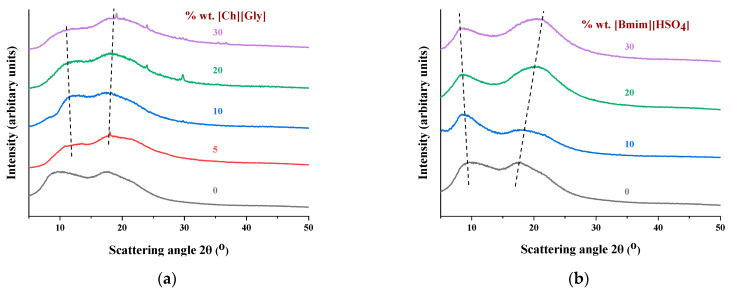
XRD patterns of CA-IL blends: (**a**) CA-$[{\mathrm{Ch}}^{+}]\left[{\mathrm{Gly}}^{-}\right]$ and (**b**) CA-$\left[{\mathrm{Bmim}}^{+}\right]$[${H\mathrm{SO}}_{4}^{-}$]. The numbers denote the % wt. IL in composite membranes. The dashed lines act as a visual guide.

The X-ray diffraction patterns of the CA composite membranes and the reference membrane of the pure polymer are presented in Figure 1. Pure CA presented two main peaks located at 2θ values of 8 and 17 degrees, related to the crystalline and amorphous phases, respectively [15,44,66,67]. More specifically, the broad peak at 2θ values of approximately 8 degrees is considered the principal characteristic of the semicrystalline acetylated derivative cellulose [66]. It corresponds to an interplanar distance of 11.1 Ǻ, which is higher than the distance of 6.13 Ǻ observed for neat cellulose due to the disorder induced by the acetylation [68,69].

As shown in Figure 1a, the crystalline peak of the $[{\mathrm{Ch}}^{+}]\left[{\mathrm{Gly}}^{-}\right]$-containing membranes was observed at a slightly higher 2θ compared to that of the neat polymer, while the amorphous halo was located almost at the same angle. Also, upon addition of $[{\mathrm{Ch}}^{+}]\left[{\mathrm{Gly}}^{-}\right]$, the amorphous halo became dominant, indicating that $[{\mathrm{Ch}}^{+}]\left[{\mathrm{Gly}}^{-}\right]$ significantly reduced the CA crystallinity. In the case of $\left[{\mathrm{Bmim}}^{+}\right]$[${H\mathrm{SO}}_{4}^{-}$], the crystalline peak at 8 degrees shifted to a slightly lower value, while the amorphous halo at 17 degrees shifted to a higher value. The ratio of those two areas remained almost constant, with an exception at 10% IL loading, in which the crystalline peak was dominant. These findings suggest that the addition of $\left[{\mathrm{Bmim}}^{+}\right]$[${H\mathrm{SO}}_{4}^{-}$] had no significant effect on the crystallinity of the CA, with an exception at 10%. Such qualitative observations are better analyzed by the estimation of the crystalline/total area ratio, which is indicative of the overall crystallinity, as presented below.

**Figure 2:**
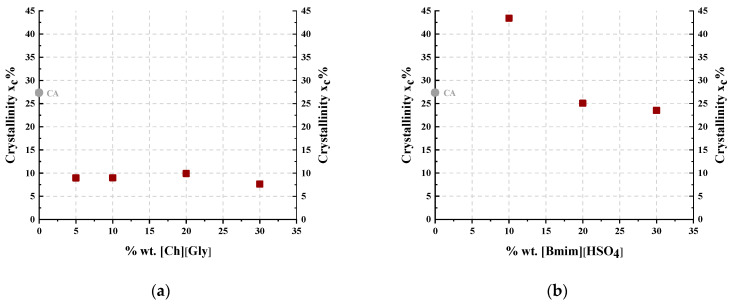
Degree of crystallinity as a function of IL content for CA-IL blends: (**a**) CA-$[{\mathrm{Ch}}^{+}]\left[{\mathrm{Gly}}^{-}\right]$ and (**b**) CA-$\left[{\mathrm{Bmim}}^{+}\right]$[${H\mathrm{SO}}_{4}^{-}$].

Table 2 summarizes the degree of crystallinity (x_c_) of the CA-IL films using a procedure described in detail in the Supplementary Information file (Section S5). Figure 2 presents the degree of crystallinity as a function of the IL content for the composite membranes. In more detail, as shown in Figure 2a, the addition of $[{\mathrm{Ch}}^{+}]\left[{\mathrm{Gly}}^{-}\right]$, even at the lowest investigated content of 5% wt., clearly decreased the CA crystallinity. Furthermore, as presented in Figure 2b for the $\left[{\mathrm{Bmim}}^{+}\right]$[${H\mathrm{SO}}_{4}^{-}$]-containing membrane, the crystallinity increased upon addition of 10% wt. $\left[{\mathrm{Bmim}}^{+}\right]$[${H\mathrm{SO}}_{4}^{-}$], while it decreased with further addition of IL. Similar behavior has been reported for cellulose triacetate membranes doped with imadazolium-based ILs [44]. This can be attributed to two antagonistic phenomena. Firstly, the addition of the IL facilitated chain mobility, rendering their reorientation more feasible and consequently favoring crystallization. For this reason, the CA membrane containing 10% wt. $\left[{\mathrm{Bmim}}^{+}\right]$[${H\mathrm{SO}}_{4}^{-}$] presented increased crystallinity compared to the neat CA. However, at the same time, the addition of IL resulted in the dilution of polymer chains, facilitating the destruction of the crystal structure. The results revealed that upon addition of more than 10% wt. $\left[{\mathrm{Bmim}}^{+}\right]$[${H\mathrm{SO}}_{4}^{-}$], the latter effect was the dominant one, leading to a decrease in crystallinity. Furthermore, as presented in Figure 2a, the addition of $[{\mathrm{Ch}}^{+}]\left[{\mathrm{Gly}}^{-}\right]$, even at the lowest investigated content of 5% wt., clearly decreased the CA crystallinity. These findings suggest that $[{\mathrm{Ch}}^{+}]\left[{\mathrm{Gly}}^{-}\right]$ is significantly more effective than $\left[{\mathrm{Bmim}}^{+}\right]$[${H\mathrm{SO}}_{4}^{-}$] in disrupting the CA crystal structure, probably due to the more pronounced capability of glycine to form strong, specific intermolecular interactions, e.g., hydrogen bonds, with CA groups.

#### 3.1.2. FTIR Analysis

**Figure 3:**
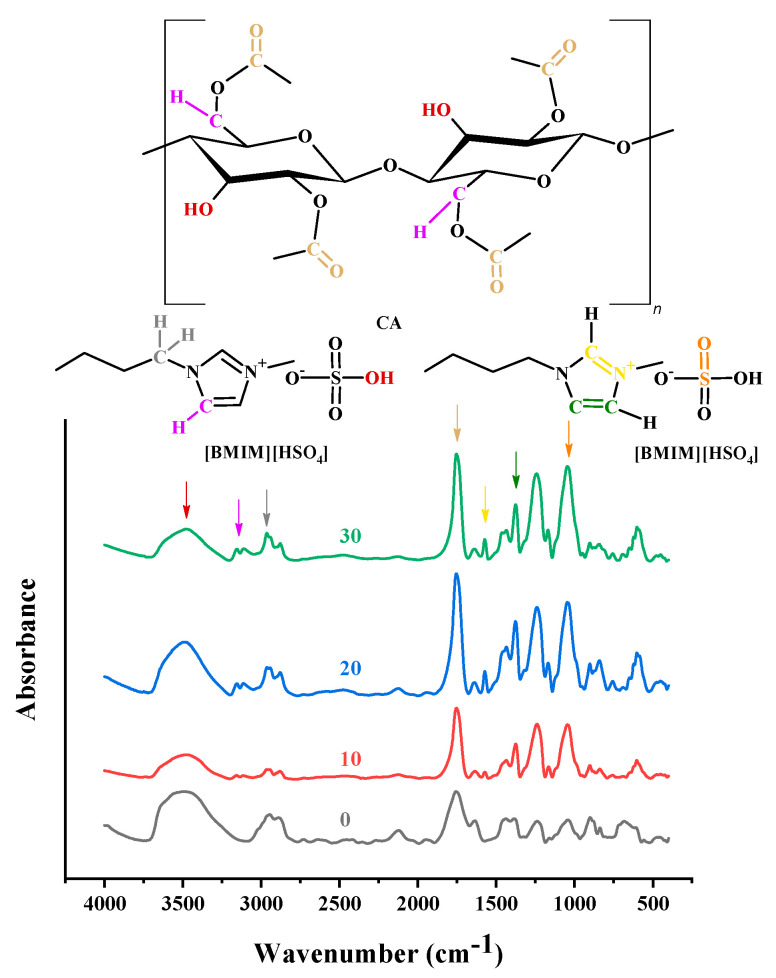
FTIR spectra of CA-$\left[{\mathrm{Bmim}}^{+}\right]$[${H\mathrm{SO}}_{4}^{-}$] membranes (with 0, 10, 20, 30% wt. $\left[{\mathrm{Bmim}}^{+}\right]$[${H\mathrm{SO}}_{4}^{-}$]) in the region 4000–400 cm^−1^.

To identify the effect of added ILs on the CA matrix, IR spectroscopy measurements were performed for CA-doped with $\left[{\mathrm{Bmim}}^{+}\right]$[${H\mathrm{SO}}_{4}^{-}$] or $[{\mathrm{Ch}}^{+}]\left[{\mathrm{Gly}}^{-}\right]$, and the obtained spectra were compared to those of pure CA. However, for the CA-$[{\mathrm{Ch}}^{+}]\left[{\mathrm{Gly}}^{-}\right]$ membranes, most likely due to the rather high water content of $[{\mathrm{Ch}}^{+}]\left[{\mathrm{Gly}}^{-}\right]$, it was not possible to obtain a spectrum of good quality. Nevertheless, regarding the $\left[{\mathrm{Bmim}}^{+}\right]$[${H\mathrm{SO}}_{4}^{-}$]-containing membranes, very interesting conclusions were obtained. In more detail, as shown in Figure 3, the pure CA membrane spectrum presented FTIR peaks corresponding to C-H stretching at around 2900 cm^−1^, O-H stretching in the region of 3700–3200 cm^−1^, and carbonyl stretching at around 1750 cm^−1^ [70]_._ Upon addition of $\left[{\mathrm{Bmim}}^{+}\right]$[${H\mathrm{SO}}_{4}^{-}$], two new peaks, attributed to the IL, were observed: a characteristic C-H stretch double peak at 3200–3000 cm^−1^ and a characteristic C=N vibration peak at 1575 cm^−1^. The other three characteristic peaks of $\left[{\mathrm{Bmim}}^{+}\right]$[${H\mathrm{SO}}_{4}^{-}$] at 3000–2900 cm^−1^ (C–H stretch), 1431 cm^−1^ (C=C stretch), and 1055 cm^−1^ (S=O bending) [42] overlapped with CA bands.

**Figure 4:**
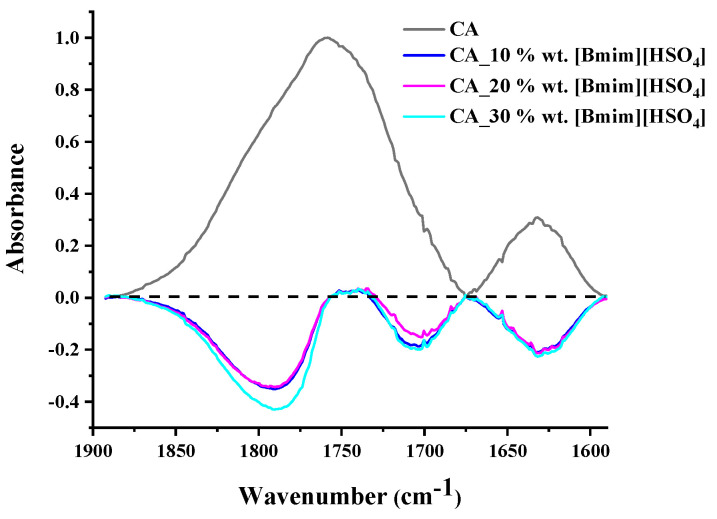
CA spectrum and subtracted spectra of the blend membranes in the region 1900–1590 cm^−1^ (the CA spectrum was subtracted from the blend membrane spectra).

It is known that CA suffers from the vinegar syndrome (acetic acid is produced due to the hydrolysis of some acetate groups) [71]. The existence of free acid and water within the CA membrane renders the evaluation of the O-H stretching region quite complex. For this reason, in order to explore any interactions between CA and $\left[{\mathrm{Bmim}}^{+}\right]$[${H\mathrm{SO}}_{4}^{-}$], only the region of the carbonyl stretching vibration was examined. In Figure 4, the CA spectrum along with the subtracted spectra of the $\left[{\mathrm{Bmim}}^{+}\right]$[${H\mathrm{SO}}_{4}^{-}$]-containing membranes (blend membrane spectrum minus CA spectrum) are presented in the region of 1900–1590 cm^−1^. As can be observed, CA exhibited two bands: one at around 1630 cm^−1^, which is attributed to water bending vibration, and one at around 1760 cm^−1^, which is attributed to C=O stretching. In the subtracted spectra, various negative peaks were observed. From the negative peak at 1630 cm^−1^, it can be concluded that the blend membranes exhibited a lower water content than pure CA. A negative peak at around 1710 cm^−1^ was also observed. The absorption in this region is typically attributed to the acid carbonyl group. Thus, the negative peak at 1710 cm^−1^ could be attributed to the lower free acetic acid content of the blend membranes. Before proceeding, it must be recalled that strong, specific intermolecular interactions can weaken the chemical bonds and alter their force constant, resulting in a decrease in the vibration frequency of the bond [72]. The negative peak at around 1780 cm^−1^ points out that the CA-free C=O groups decreased in the blend membranes. This suggests that the C=O groups of CA were influenced by the presence of IL and were most likely involved in strong intermolecular interactions with IL groups. This is reasonable for the used CA sample (with a high degree of substitution (DS) of 2.45). More precisely, the high DS of CA translated to a high C=O to OH ratio. In other words, there were not enough OH groups to strongly bind with all the C=O groups. The addition of IL provided an excess of groups that could strongly interact with the C=O groups. Thus, it is reasonable to find that the number of free C=O groups in the blend membranes decreased compared to that of neat CA. This is an important observation and can be considered for interpreting the thermal analysis and sorption results.

#### 3.1.3. Thermal Behavior

**Figure 5:**
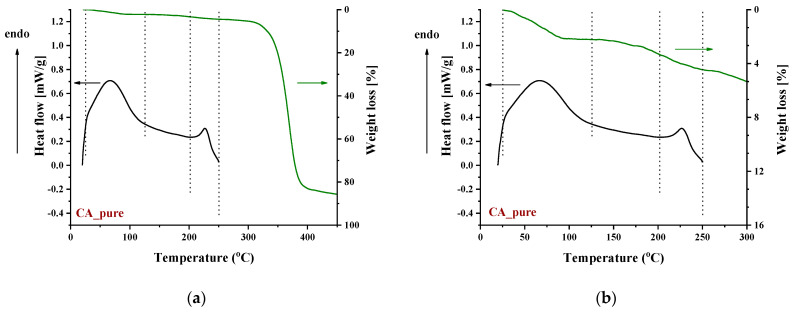
DSC and TGA curves of pure CA in the temperature range: (**a**) 0–450 °C and (**b**) 0–300 °C.

Recently, some new insights on the thermal behavior of CA have been reported [72,73,74], and it was shown that the thermal behavior of CA is not the typical one that involves thermophysical transitions, i.e., glass transition, melting point, and simple evaporation of impurities. On the contrary, the thermal behavior of CA was reported to be characterized by various peculiarities. CA exhibits similar effects to those of thermoplastic polymers, e.g., softening and evaporation of impurities; however, all these effects are of a thermochemical nature, that is, alteration of the chemical structure of CA occurs during softening or vaporization of impurities. More precisely, the broad endothermic peak at around 100 °C in the DSC curve of CA arose mainly from the enthalpy of esterification (the free acid upon heating reacted with the OH groups of CA, and water was produced) and some water evaporation (Figure 5). In other words, CA contains free acetic acid (the acetic acid may be a residue of the preparation of CA or is produced by the hydrolysis of acetate groups). By heating, the free acetic acid esterifies the free OH groups of CA, and water is produced. Similarly, the endodermic peak observed in the first DSC scan of CA around 230 °C was not due to neat melting but rather to simultaneous softening and decomposition (Figure 5). The term “*thermochemical transition*” was proposed to describe this effect (simultaneous softening and decomposition), and more recently, it was recognized that this is just a special case of a more general property named “melting inability” [75]. Also, recently, it was reported that substances with an increased number of hydrogen-bonded groups, such as gallic acid and quercetin, cause a depression of the thermochemical transition temperature of CA [72]. This was explained based on the weakening of the chemical bonds due to the formation of strong intermolecular interactions between the additive (e.g., gallic acid) and CA. The addition of such substances in CA, as mentioned above for the case of IL, provides an excess of groups to interact with the C=O (or other groups) of CA. Thus, the thermal behavior of pure CA will not be further discussed.

**Figure 6:**
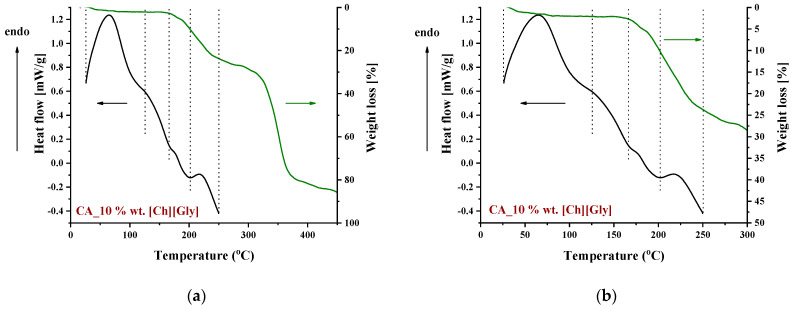
DSC and TGA curves of a CA membrane doped with 10% wt. $[{\mathrm{Ch}}^{+}]\left[{\mathrm{Gly}}^{-}\right]$ in the temperature range: (**a**) 0–450 °C and (**b**) 0–300 °C.

**Figure 7:**
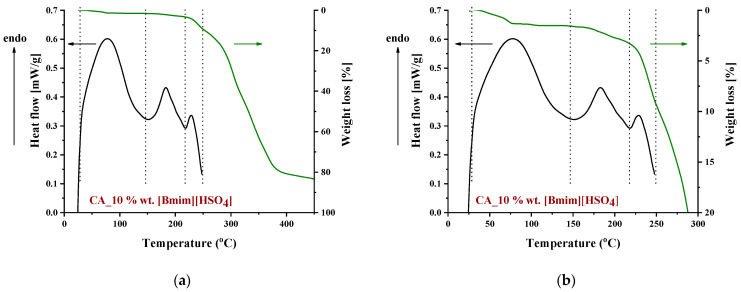
DSC and TGA curves of a CA membrane doped with 10% wt. $\left[{\mathrm{Bmim}}^{+}\right]$[${H\mathrm{SO}}_{4}^{-}$] in the temperature range: (**a**) 0–450 °C and (**b**) 0–300 °C.

**Figure 8:**
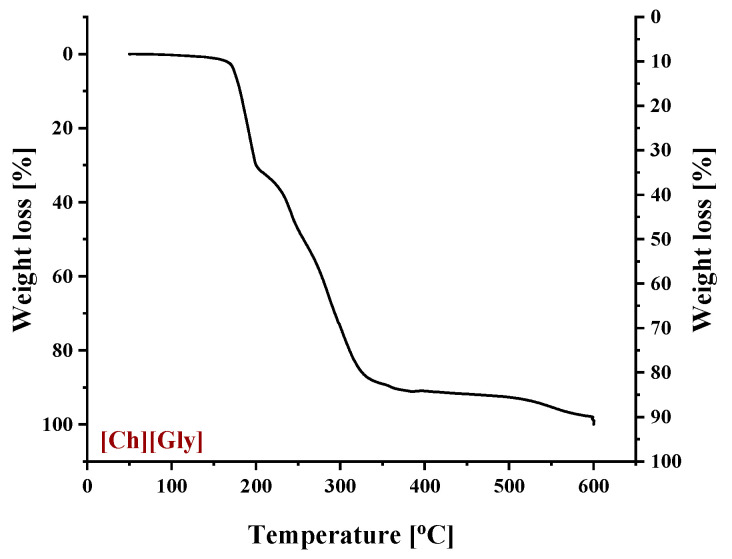
Thermogravimetric curve of pure $[{\mathrm{Ch}}^{+}]\left[{\mathrm{Gly}}^{-}\right]$.

**Figure 9:**
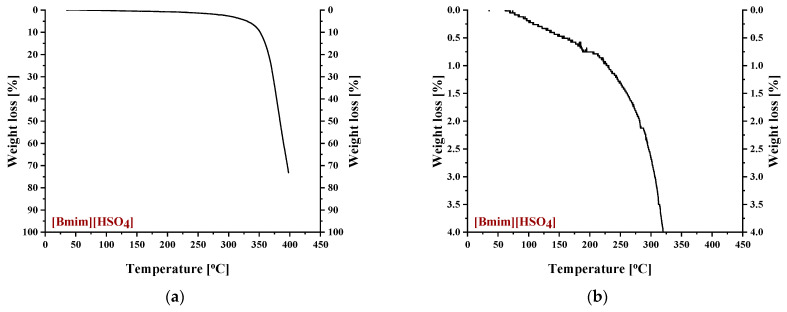
Thermogravimetric curves of pure $\left[{\mathrm{Bmim}}^{+}\right]$[${H\mathrm{SO}}_{4}^{-}$] for weight loss range: (**a**) 0–100% and (**b**) 0–4%.

To identify the thermal events taking place upon heating in the composite CA-IL matrix, the DSC and TGA heating curves of CA membranes containing 10% wt. $[{\mathrm{Ch}}^{+}]\left[{\mathrm{Gly}}^{-}\right]$ and 10% wt. $\left[{\mathrm{Bmim}}^{+}\right]$[${H\mathrm{SO}}_{4}^{-}$] are presented in Figure 6 and Figure 7, respectively. In both cases (Figure 6 and ), the DSC curves of the CA doped with IL membranes showed a broad endothermic peak up to 125 °C corresponding to the evaporation of IL and CA impurities as well as evaporation of water produced by esterification, in agreement with the mass loss of 2.5% wt. shown in the TGA curves. However, the thermal behavior of the two composites differed with a further increase in temperature. In more detail, the DSC curve of CA doped with $[{\mathrm{Ch}}^{+}]\left[{\mathrm{Gly}}^{-}\right]$ membrane presented two events, one around 130 °C and another around 175 °C, while the DSC curve of CA doped with $\left[{\mathrm{Bmim}}^{+}\right]$[${H\mathrm{SO}}_{4}^{-}$] membrane showed only one significant thermal event around 175 °C. It should be noted that the event for $[{\mathrm{Ch}}^{+}]\left[{\mathrm{Gly}}^{-}\right]$-containing membranes is not associated with mass loss, as shown by the respective TGA curve. Such behavior was also observed by Lam et al. [44] for cellulose triacetate (CTA) membranes containing imidazolium-based ILs, and it was attributed to the glass transition of the polymer. However, recently, some new insights into the thermal behavior of CA have been reported [72,73,74]. Also, in this work, acetic acid was used as a solvent for the membrane preparation, and thus, acetic acid residue would be expected due to the strong intermolecular interactions with the membrane’s constituents. The presence of carboxyl groups may interfere with the esterification reaction between free acetic acid and the OH groups of CA (CTA normally should not have any free OH). The thermal effect around 130 °C (see Figure 6) began to occur at a temperature very close to the boiling point of acetic acid (118 °C). Based on the above, this signal alteration seems to be related to acetic acid evaporation. The fact that no mass loss was detected in TGA was simply because it was lower than the detection limit. In any case, multiple effects took place, and for the explanation of this phenomenon, further investigation is needed. Furthermore, the endothermic DSC peaks that were observed around 175 °C were associated with approximately 10% and 2.5% wt. mass loss for the $[{\mathrm{Ch}}^{+}]\left[{\mathrm{Gly}}^{-}\right]$ and $\left[{\mathrm{Bmim}}^{+}\right]$[${H\mathrm{SO}}_{4}^{-}$]-containing membranes, respectively. Such mass loss is presumably attributed to the decomposition of the IL-rich regions because, in this temperature range, the decomposition of pure $[{\mathrm{Ch}}^{+}]\left[{\mathrm{Gly}}^{-}\right]$ (Figure 8) and the partial decomposition of $\left[{\mathrm{Bmim}}^{+}\right]$[${H\mathrm{SO}}_{4}^{-}$] (Figure 9) occur. Finally, for both membranes, the thermochemical transition of CA occurred around 230 °C, which is associated with mass loss steps of 15% and 5% wt. for the CA-$[{\mathrm{Ch}}^{+}]\left[{\mathrm{Gly}}^{-}\right]$ and the CA-$\left[{\mathrm{Bmim}}^{+}\right]$[${H\mathrm{SO}}_{4}^{-}$] membranes, respectively.

**Figure 10:**
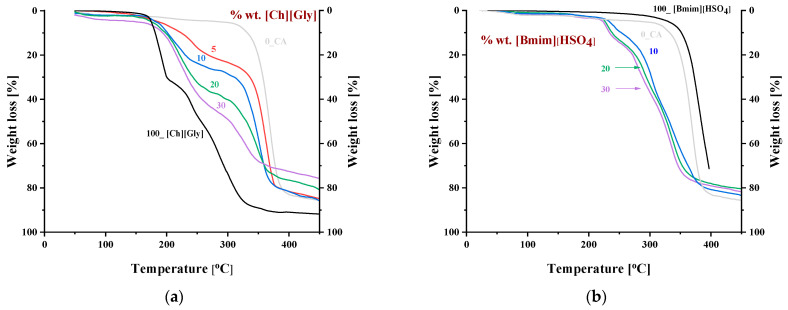
Thermogravimetric curves of CA doped with (**a**) $[{\mathrm{Ch}}^{+}]\left[{\mathrm{Gly}}^{-}\right]$ and (**b**) $\left[{\mathrm{Bmim}}^{+}\right]$[${H\mathrm{SO}}_{4}^{-}$]. The numbers denote the % wt. IL content in the composite membranes.

Figure 10 shows the thermogravimetric (TGA) curves of CA-IL composite membranes at a heating rate of 10 °C min^−1^. It was observed that the degradation temperature of CA decreased with increasing IL content. This decrease was higher for the CA-$[{\mathrm{Ch}}^{+}]\left[{\mathrm{Gly}}^{-}\right]$ blends, indicating that $[{\mathrm{Ch}}^{+}]\left[{\mathrm{Gly}}^{-}\right]$ was more effective than $\left[{\mathrm{Bmim}}^{+}\right]$[${H\mathrm{SO}}_{4}^{-}$] in CA polymer chain disruption, lowering the polymer chain bonding energy and subsequently increasing their mobility. This is expected due to the increased strong molecular interactions that can be formed between glycine and CA. This was also confirmed by the significant decrease in crystallinity (Figure 2) observed for $[{\mathrm{Ch}}^{+}]\left[{\mathrm{Gly}}^{-}\right]$ blends.

**Figure 11:**
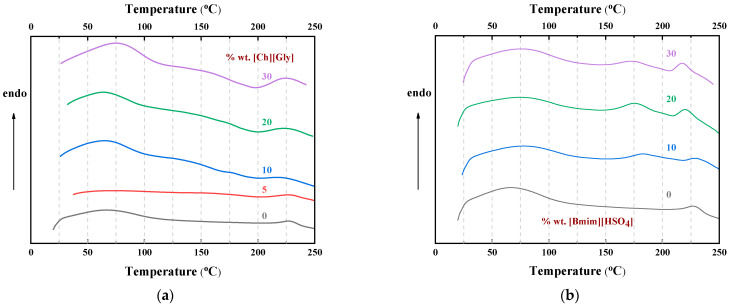
DSC heat flow curves of CA-IL blends obtained at a heating rate of 10 °C/min for (**a**) $[{\mathrm{Ch}}^{+}]\left[{\mathrm{Gly}}^{-}\right]$ and (**b**) $\left[{\mathrm{Bmim}}^{+}\right]$[${H\mathrm{SO}}_{4}^{-}$]. The numbers denote the % wt. IL content in the composite membranes.

The DSC curves obtained by heating up to 250 °C using a heating rate of 10 °C min^−1^ for the CA-IL blends are shown in Figure 11. As can be seen, in all cases, the thermochemical transition temperature of CA was depressed. Also, a similar depression could be observed for the temperature related to the decomposition temperature of IL (around 175 °C). Such observation is in agreement with the TGA results and, based on the abovementioned FTIR discussion, can be explained by keeping in mind the weakening of the chemical bond strength due to the strong intermolecular interactions.

### 3.2. CO_2_ Sorption Measurements in CA-IL Membranes

The sorption of N_2_ was too low to be measured by the adopted method. Until the sample could be transferred to the scale, desorption occurred, and any detectable mass loss was very close to the scale’s readability (0.0001 g). Thus, only the results for CO_2_ sorption will be presented. Clearly, the above shows that the membranes exhibit selectivity for CO_2_ compared to N_2_; however, this selectivity cannot be quantified due to the lack of data for the N_2_ sorption.

**Figure 12:**
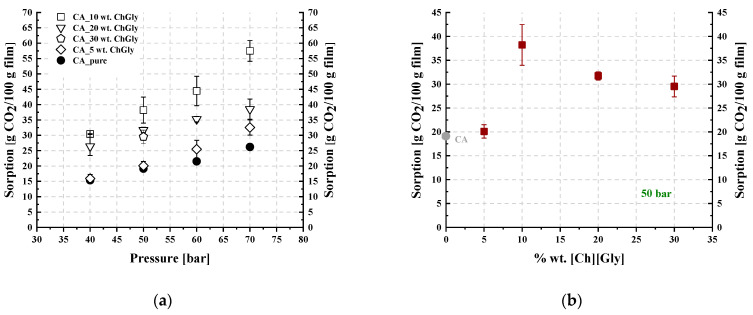
(**a**) CO_2_ sorption isotherms at 35 °C and (**b**) trend of CO_2_ sorption as a function of the IL content at 50 bar and 35 °C, for CA-$[{\mathrm{Ch}}^{+}]\left[{\mathrm{Gly}}^{-}\right]$ blends.

**Figure 13:**
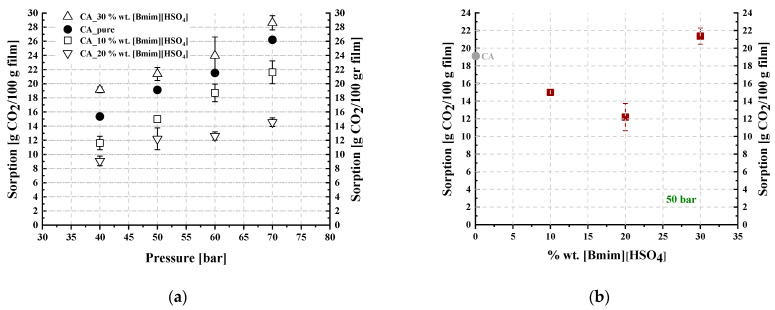
(**a**) CO_2_ sorption isotherms at 35 °C and (**b**) trend of CO_2_ sorption as a function of the IL content at 50 bar and 35 °C, for CA-$\left[{\mathrm{Bmim}}^{+}\right]$[${H\mathrm{SO}}_{4}^{-}$] blends.

Table 3 and Table 4 summarize the CO_2_ sorption results for the studied membranes, which were obtained at 35 °C and in the pressure range of 50 to 70 bar. The results are illustrated in Figure 12 and Figure 13, where the CO_2_ sorption isotherms and the effect of the IL content on sorption are presented for all investigated membranes. The results of the pure CA membrane were in agreement with those reported in the literature [15]. The IL loading effect on CO_2_ sorption was more apparent with increasing pressure (Figure 12a and Figure 13a).

As shown in Figure 12a, all investigated $[{\mathrm{Ch}}^{+}]\left[{\mathrm{Gly}}^{-}\right]$-containing membranes presented increased CO_2_ sorption compared to that of neat CA. However, as presented in Figure 12b, sorption did not change monotonically when plotted against the IL content, with a maximum observed for the 10% wt. $[{\mathrm{Ch}}^{+}]\left[{\mathrm{Gly}}^{-}\right]$-containing membrane. A similar observation was reported by Reed et al. [65], who revealed increasing CO_2_ solubility in cellulose membranes containing a solid ammonium-based organic salt, i.e., tetraethyl ammonium acetate, for up to 25% wt. of salt content and a subsequent decrease in CO_2_ solubility by increasing the salt content to 50% wt. In other words, they observed a maximum CO_2_ solubility similar to the maximum presented in Figure 12b.

Furthermore, as presented in Figure 13, the addition of $\left[{\mathrm{Bmim}}^{+}\right]$[${H\mathrm{SO}}_{4}^{-}$] in the CA membrane up to 20% wt. reduced the overall sorption ability per membrane unit mass, while further addition of $\left[{\mathrm{Bmim}}^{+}\right]$[${H\mathrm{SO}}_{4}^{-}$] to 30% wt. resulted in an increase in CO_2_ dissolution. Thus, minimum CO_2_ sorption was observed at 20% wt. $\left[{\mathrm{Bmim}}^{+}\right]$[${H\mathrm{SO}}_{4}^{-}$] content. Such behavior was also observed by Lam et al. [44], who reported decreasing CO_2_ solubility in cellulose triacetate membranes containing an imidazolium-based IL, i.e., 1-ethyl-3-methylimidazolium dicyanamide, for up to 40% wt. IL content and a subsequent increase in CO_2_ solubility by increasing the IL content to 50% wt. In other words, they observed a minimum CO_2_ solubility similar to the minimum presented in Figure 13b.

**Figure 14:**
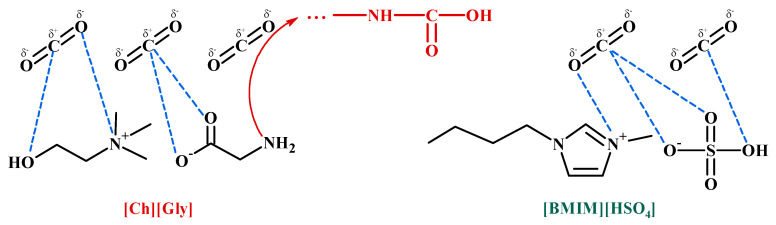
Main intermolecular (dashed lines) and chemical interactions (red line) of CO_2_ with $[{\mathrm{Ch}}^{+}]\left[{\mathrm{Gly}}^{-}\right]$ and $\left[{\mathrm{Bmim}}^{+}\right]$[${H\mathrm{SO}}_{4}^{-}$].

Let us first comment on the strong CO_2_-IL intermolecular interactions. As mentioned in the introduction section, the interaction of CO_2_ (Lewis acid, LA) with the anion of ILs (Lewis base, LB) is an important factor for determining CO_2_ solubility in ILs. It was shown that there are two contributing factors to the relative high CO_2_ solubility in 1-ethyl-3-methylimidazolium hydrogen sulfate ($\left[{\mathrm{Emim}}^{+}\right]$[${H\mathrm{SO}}_{4}^{-}$]): the high sulfonyl group (S=O) polarization, which leads to stronger LA-LB interactions with CO_2_ [56], and the high negative charge of all oxygen atoms in the HSO_4_^−^ anion, resulting in strong polar interactions with the positively charged carbon atom of CO_2_ [57]. Furthermore, the interaction of CO_2_ with the positively charged nitrogen atom in the imidazolium ring or the cation of choline is expected to be less strong due to the sterical hindrance imposed by the alkyl groups connected to nitrogen. Such intermolecular interactions are schematically presented in Figure 14.

However, the existence of extrema (minimum or maximum) of CO_2_ sorption reveals antagonistic phenomena. Nevertheless, only the strong and/or chemical interactions of CO_2_-IL cannot explain the CO_2_ sorption behavior because the competitive CA-CO_2_ and CA-IL intermolecular interactions are also important.

In more detail, Kazarian [76], using an FTIR analysis, showed that polymers containing >C=O groups presented significantly higher CO_2_ sorption ability due to the rather strong LA-LB interactions between the positively charged carbon atom of CO_2_ and the oxygen of the carbonyl group. Consequently, the sorption of CO_2_ increased with the increasing number of >C=O groups in the polymer chains.

**Figure 15:**
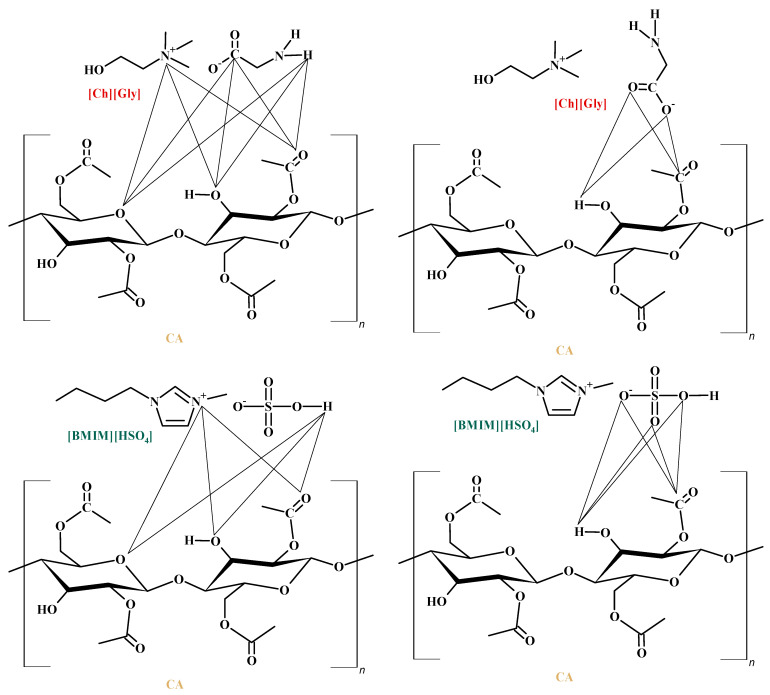
Main CA-ILs with strong intermolecular interactions.

However, the addition of ILs inside the polymer matrix, which presents a lot of groups that can strongly interact with >C=O groups of the polymer chain, reduces the available (unbound) carbonyl groups for interaction with CO_2_, thereby reducing the ability of CA to dissolve CO_2_. This is in accordance with the experimental DSC/TGA and FTIR observations. Specifically, as discussed in the previous section, the thermal analysis showed a depression of the thermochemical transition temperature of the IL-containing membranes, an observation that can be attributed to increased IL-CA intermolecular interactions. Furthermore, the FTIR results presented in a previous section showed a reduction in the unbound >C=O groups of the polymer that are available for interaction with CO_2_. Some important polymer–IL interactions for both the investigated ILs are shown in Figure 15.

Nevertheless, as discussed above and shown in Figure 14, the addition of ILs not only reduces the available (unbound) polymer groups for interaction with CO_2_, thus tending to reduce the overall CO_2_ sorption, but, at the same time, introduces new sites on the IL ions that can strongly interact with CO_2_, thereby favoring the sorption of the gas inside the composite membrane matrix. Thus, upon the addition of an IL to the polymer membrane, there is an interplay of favorable CA-IL interactions, acting competitively with the favorable CO_2_-CA and CO_2_-IL intermolecular interactions. The net effect of such competing phenomena is the existence of extrema when sorption is plotted against the IL content.

More specifically, as shown in Figure 12b, upon addition of $[{\mathrm{Ch}}^{+}]\left[{\mathrm{Gly}}^{-}\right]$ to the CA membranes up to 10% wt., the introduction of -NH_2_ groups that are capable of CO_2_ chemical absorption and the introduction of the rest of the IL sites that are capable of strong physical intermolecular interactions with CO_2_ were the dominating factors resulting in the increase in the overall CO_2_ dissolution in the membrane. At $[{\mathrm{Ch}}^{+}]\left[{\mathrm{Gly}}^{-}\right]$ contents higher than 20% wt., the reduction in the polymer groups that are capable of strong interactions with CO_2_ dominated, resulting in a small decrease in CO_2_ solubility and the appearance of a maximum in the plot (Figure 12b).

On the other hand, as shown in Figure 13b, upon addition of $\left[{\mathrm{Bmim}}^{+}\right]$[${H\mathrm{SO}}_{4}^{-}$] in the CA membranes up to 20% wt., the reduction in the polymer groups that are capable of strong intermolecular interactions with CO_2_ had a more severe effect than the introduction of new IL sites, causing a reduction in the overall CO_2_ solubility in the membrane. Further addition of $\left[{\mathrm{Bmim}}^{+}\right]$[${H\mathrm{SO}}_{4}^{-}$] up to 30% wt. was translated to the addition of new IL sites, increasing CO_2_ solubility and resulting in the appearance of a minimum in the plot of Figure 13b.

In conclusion, the CO_2_ sorption behavior of CA-IL composite membranes cannot be explained only by strong CO_2_-IL intermolecular or/and chemical interactions, and CA-CO_2_ and CA-IL intermolecular interactions must also be considered. In general, CO_2_ sorption in low-molecular-weight systems, such as ionic liquids, is affected by the presence of a third component [77,78] (CA in this case).

As mentioned above, it is known [76] that polymers with C=O groups exhibit increased CO_2_ sorption capability. The solubility of CO_2_ in the pure CA membranes that were developed in this study (solubility at 35 °C and 50 bar equal to 19.1 g CO_2_/100 g polymer) was similar or higher than that in other polymers with C=O groups, e.g., poly(methyl methacrylate) (solubility at 35 °C and 50 bar equal to 12.5 g CO_2_/100 g polymer [79]) and almost triple than the solubility in polymers without C=O groups, e.g., poly(styrene) (solubility at 35 °C and 50 bar equal to 7 g CO_2_/100 g polymer [79]). For cellulose triacetate at the same temperature but lower pressures, e.g., 13 bar, the solubility has been reported to be 6 g CO_2_/100 g polymer [44]. At 35 °C and 20 bar, the solubility in polycarbonate has been reported to be 5 g CO_2_/100 g polymer [80]. For PPO (poly(2,6-dimethyl-1,4-phenylene ether)) at similar pressures as those used in this study (40 bar) but at higher temperatures, e.g., 100 °C, the solubility has been reported to be 5 g CO_2_/100 g polymer, while at the same temperature but considerably higher pressure (200 bar), the solubility increases to 17.5 g CO_2_/100 g polymer [81], that is, slightly lower than the solubility of CO_2_ in the CA membrane measured at 35 °C and 50 bar. Also, in the literature, cellulose triacetate–IL membranes have been found to exhibit a behavior similar to the studied CA-$\left[{\mathrm{Bmim}}^{+}\right]$[${H\mathrm{SO}}_{4}^{-}$] membranes [44]. Finally, the studied CA-$[{\mathrm{Ch}}^{+}]\left[{\mathrm{Gly}}^{-}\right]$ membranes with 10% IL exhibited practically double solubility values compared to the pure CA membranes, which, as just discussed, exhibited high solubility compared to other polymers. Thus, the CA-10%$[{\mathrm{Ch}}^{+}]\left[{\mathrm{Gly}}^{-}\right]$ membrane has a great potential for CO_2_ sorption as it exhibits higher solubility compared to other available polymeric materials.

In Table 5 and Table 6, the diffusion coefficients (D) of CO_2_ in the studied membranes are presented. It should be stressed that the calculation of D by the adopted procedure, though it is common in the literature, is based on various assumptions, e.g., Fickian desorption, D being independent of concentration, etc. Thus, the presented values of D cannot be considered accurate values; nevertheless, useful conclusions can be extracted by comparing the order of magnitude of these values. As can be seen in Table 5, the addition of 5% $[{\mathrm{Ch}}^{+}]\left[{\mathrm{Gly}}^{-}\right]$ in CA increased the diffusion coefficient by one order of magnitude, while at higher concentrations, the increase was by two orders of magnitude. These values are comparable with the literature values for polymer–IL membranes [43]. The value of D for pure CA was lower compared to the ones of polymers with C=O groups, e.g., PMMA [82]. An explanation for this could be that, in a polymer like PMMA, plasticization occurs during sorption, while sorption occurs faster in the rubbery state than in the glassy state [82]. In any case, the values of D further support the high potential and the beneficial effect of $[{\mathrm{Ch}}^{+}]\left[{\mathrm{Gly}}^{-}\right]$ as the addition of $[{\mathrm{Ch}}^{+}]\left[{\mathrm{Gly}}^{-}\right]$ results in not only increased equilibrium concentration but also faster sorption. However, this is not the case for the CA-$\left[{\mathrm{Bmim}}^{+}\right]$[${H\mathrm{SO}}_{4}^{-}$] membranes, as can be seen in Table 6. In the values of D of the CA-$\left[{\mathrm{Bmim}}^{+}\right]$[${H\mathrm{SO}}_{4}^{-}$] membranes, some increasing tendency could be observed, but the composite membranes exhibited only slightly higher values of D and of the same order of magnitude as the ones of pure CA. The addition of IL, which is a low-molecular-weight substance, to a polymer would be expected to increase the mobility of the macromolecules and increase the free volume of the polymer to some extent (such effects are very intense during plasticization). Thus, in the presence of small molecules, the CO_2_ molecules can diffuse faster inside the polymer network. This mechanism contributed to the increase in D in both cases. However, such effects are expected to be more intense in the $[{\mathrm{Ch}}^{+}]\left[{\mathrm{Gly}}^{-}\right]$-containing membranes because the interactions of CA with $[{\mathrm{Ch}}^{+}]\left[{\mathrm{Gly}}^{-}\right]$ is stronger than those of CA with $\left[{\mathrm{Bmim}}^{+}\right]$[${H\mathrm{SO}}_{4}^{-}$]. In addition, in the case of $[{\mathrm{Ch}}^{+}]\left[{\mathrm{Gly}}^{-}\right]$ and due to its intermolecular interactions with CO_2_, an additional mechanism for the diffusion of CO_2_ is present, and for these reasons, the increase in D was much higher in the corresponding $[{\mathrm{Ch}}^{+}]\left[{\mathrm{Gly}}^{-}\right]$-containing membranes.

A similar increase can be observed in the permeability values of the CA-$[{\mathrm{Ch}}^{+}]\left[{\mathrm{Gly}}^{-}\right]$ membranes (Table 7). More specifically, the permeability of CA increased by one or two orders of magnitude, depending on the amount of $[{\mathrm{Ch}}^{+}]\left[{\mathrm{Gly}}^{-}\right]$. It is worth mentioning that these values were higher than the values of other polymer–IL membranes, despite the fact that the latter might exhibit higher D values [43]. This is due to the high CO_2_ solubility in the $[{\mathrm{Ch}}^{+}]\left[{\mathrm{Gly}}^{-}\right]$-containing membranes. It should be recalled that the permeability is proportional to the solubility and the diffusion coefficient. By keeping this in mind, the permeability results for the CA-$\left[{\mathrm{Bmim}}^{+}\right]$[${H\mathrm{SO}}_{4}^{-}$] membranes (Table 8) can be understood. The solubility of the composite membranes was lower; however, the slightly increased D values resulted in a (slight) increase in the permeability values, which was comparable to the literature values [43].

## 4. Conclusions

Two different ILs ($\left[{\mathrm{Bmim}}^{+}\right]$[${H\mathrm{SO}}_{4}^{-}$] and $[{\mathrm{Ch}}^{+}]\left[{\mathrm{Gly}}^{-}\right]$) were used for the preparation of CA-IL composite membranes. A new route was used to synthesize a biodegradable and non-toxic [Ch^+^][Gly^-^] ionic liquid. The synthesized membranes were characterized by a variety of methods, and their potential for utilization in CO_2_ separation processes was evaluated by experimental measurements of CO_2_ sorption.

The characterization results showed that the addition of ILs facilitated the chain mobility, rendering their reorientation more feasible and consequently favoring crystallization. However, at the same time, the addition of ILs resulted in the dilution of polymer chains, facilitating the destruction of the crystal structure. Such competitive phenomena resulted in a severe decrease in CA crystallinity upon addition of [Ch^+^][Gly^−^] up to 30% wt., while they resulted in the appearance of a maximum in crystallinity upon addition of 10% wt. $\left[{\mathrm{Bmim}}^{+}\right]$[${H\mathrm{SO}}_{4}^{-}$].

The FTIR analysis for the $\left[{\mathrm{Bmim}}^{+}\right]$[${H\mathrm{SO}}_{4}^{-}$]-containing CA membranes showed that the addition of IL decreased the number of free C=O groups in the blend membranes compared to the neat CA because IL provided an excess of groups that could strongly interact with the C=O groups of the polymer.

The investigation of the thermal behavior of the composite membranes showed that the $[{\mathrm{Ch}}^{+}]\left[{\mathrm{Gly}}^{-}\right]$ membranes were, in general, less thermally stable than the $\left[{\mathrm{Bmim}}^{+}\right]$[${H\mathrm{SO}}_{4}^{-}$] membranes, while the degradation temperature of CA decreased with increasing IL content. In all cases, upon the addition of IL, the thermochemical transition temperature of CA was depressed.

Upon the addition of an IL to the polymer, there was an interplay of favorable CA-IL interactions acting competitively with the favorable CO_2_-CA and CO_2_-IL intermolecular interactions. Such behavior resulted in the appearance of extrema when the CO_2_ sorption was plotted against the IL content, i.e., a maximum appeared for the $[{\mathrm{Ch}}^{+}]\left[{\mathrm{Gly}}^{-}\right]$-containing membranes and a minimum for the $\left[{\mathrm{Bmim}}^{+}\right]$[${H\mathrm{SO}}_{4}^{-}$]-containing membranes. In all cases, $[{\mathrm{Ch}}^{+}]\left[{\mathrm{Gly}}^{-}\right]$ membranes presented higher CO_2_ solubility than neat CA and $\left[{\mathrm{Bmim}}^{+}\right]$[${H\mathrm{SO}}_{4}^{-}$] membranes. The $[{\mathrm{Ch}}^{+}]\left[{\mathrm{Gly}}^{-}\right]$ membranes also exhibited significantly higher diffusion coefficients and permeability values. In other words, they not only absorbed higher amounts of CO_2_ but also absorbed these amounts at a higher rate than pure CA. In combination with the poor solubility of N_2_ in these membranes (too low to be measured), it can be concluded that these membranes exhibit selectivity and a high potential for increased and fast CO_2_ capture.

Besides these aspects, $[{\mathrm{Ch}}^{+}]\left[{\mathrm{Gly}}^{-}\right]$ exhibits additional advantages over the other ILs, such as non-toxicity, biodegradability, and the low cost of the precursor chemicals. Thus, it seems that the combination of $[{\mathrm{Ch}}^{+}]\left[{\mathrm{Gly}}^{-}\right]$ with an eco-friendly and low-cost CA polymer is a very promising approach for effective and sustainable CO_2_ capture applications.

## Figures and Tables

**Table 1:** Structure of cellulose acetate (CA), used as a polymeric base, and ionic liquids (ILs), used as liquid mediums, for the preparation of the CA-IL membranes.

Polymeric Base	
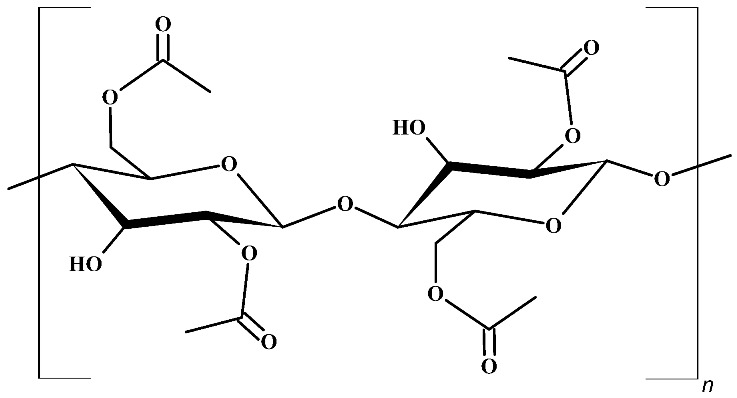 Cellulose acetate, CA	
Ionic Liquids	
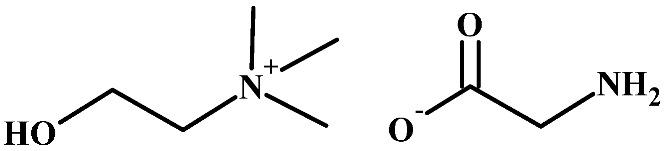 Choline glycine, $[{\mathrm{Ch}}^{+}]\left[{\mathrm{Gly}}^{-}\right]$	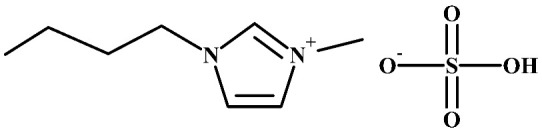 1-Butyl-3-methylimidazolium hydrogen sulfate, $\left[{\mathrm{Bmim}}^{+}\right]$[${H\mathrm{SO}}_{4}^{-}$]

**Table 2:** Degree of crystallinity (x_c_) * for CA doped with IL blends.

IL %wt.	**CA-** $[{\mathrm{Ch}}^{+}]\left[{\mathrm{Gly}}^{-}\right]$	**CA-**$\left[{\mathrm{Bmim}}^{+}\right]$$[{\mathrm{HSO}}_{4}^{-}$]
0	27	27
5	9	--
10	9	43
20	10	25
30	8	24
100	--	--

* x_c_ determined by XRD analysis.

**Table 3:** CO_2_ sorption (g CO_2_ per 100 g of film) of CA films containing $[{\mathrm{Ch}}^{+}]\left[{\mathrm{Gly}}^{-}\right]$ at 35 °C.

IL Content/	Pressure/Bar			
%wt.	40	50	60	70
0	15.4 ± 0.4	19.1 ± 0.2	21.5 ± 0.5	26.2 ± 0.2
5	15.9 ± 1.3	20.1 ± 1.4	25.5 ± 2.9	32.6 ± 2.5
10	30.4 ± 0.1	38.2 ± 4.3	44.5 ± 4.8	57.5 ± 3.4
20	26.4 ± 3.0	31.7 ± 0.9	35.3 ± 0.9	38.5 ± 3.3
30	--	29.5 ± 2.2	--	--

**Table 4:** CO_2_ sorption (g CO_2_ per 100 g of film) of CA films containing $\left[{\mathrm{Bmim}}^{+}\right]$[${H\mathrm{SO}}_{4}^{-}$] at 35 °C.

IL Content/	Pressure/Bar			
%wt.	40	50	60	70
0	15.4 ± 0.4	19.1 ± 0.2	21.5 ± 0.5	26.2 ± 0.2
10	11.6 ± 1.0	15.0 ± 0.2	18.7 ± 1.2	21.6 ± 1.6
20	9.1 ± 0.7	12.2 ± 1.5	12.6 ± 0.6	14.6 ± 0.6
30	19.1 ± 0.4	21.4 ± 0.9	24.0 ± 2.6	28.6 ± 1.0

**Table 5:** Diffusion coefficient of CO_2_ in CA films containing $[{\mathrm{Ch}}^{+}]\left[{\mathrm{Gly}}^{-}\right]$ at 35 °C.

IL Content/	D/10^−7^ cm^2^ s^−1^			
%wt.	40 Bar	50 Bar	60 Bar	70 Bar
0	0.2 ± 0.1	0.4 ± 0.2	0.2 ± 0.1	0.3 ± 0.1
5	2.0 ± 0.4	1.9 ± 0.5	2.6 ± 0.4	2.6 ± 0.7
10	10.6 ± 0.2	11.1 ± 0.6	10.5 ± 0.6	9.0 ± 1.1
20	13.0 ± 1.0	14.2 ± 0.3	12.1 ± 1.0	11.1 ± 0.7
30	--	30.2 ± 6.2	--	--

**Table 6:** Diffusion coefficient **of** CO_2_ in CA films containing $\left[{\mathrm{Bmim}}^{+}\right]$[${H\mathrm{SO}}_{4}^{-}$] at 35 °C.

IL Content/	D/10^−7^ cm^2^ s^−1^			
%wt.	40 Bar	50 Bar	60 Bar	70 Bar
0	0.2 ± 0.1	0.4 ± 0.2	0.2 ± 0.1	0.3 ± 0.1
10	0.1 ± 0.1	0.4 ± 0.1	0.4 ± 0.1	0.5 ± 0.1
20	0.2 ± 0.1	0.2 ± 0.1	0.2 ± 0.1	0.2 ± 0.1
30	0.5 ± 0.1	0.5 ± 0.1	0.6 ± 0.2	0.3 ± 0.1

**Table 7:** Permeability of CO_2_ in CA films containing $[{\mathrm{Ch}}^{+}]\left[{\mathrm{Gly}}^{-}\right]$ at 35 °C.

IL Content/	$\mathrm{Permeability}/\mathrm{Barrer}\;(1\;\mathrm{Barrer}=10^{-10}\frac{{\mathrm{cm}}^{3}\mathrm{STP}\cdot \mathrm{cm}}{{\mathrm{cm}}^{2}\cdot s\cdot \mathrm{cmHg}}$)			
%wt.	40 Bar	50 Bar	60 Bar	70 Bar
0	6.5	13.2	7.4	8.5
5	68.6	68.3	95.5	104.9
10	706.5	741.9	681.9	634.9
20	752.3	790	622.5	536.4
30	--	1564.2	--	--

**Table 8:** Permeability of CO_2_ in CA films containing $\left[{\mathrm{Bmim}}^{+}\right]$[${H\mathrm{SO}}_{4}^{-}$] at 35 °C.

IL Content/	$\mathrm{Permeability}/\mathrm{Barrer}\;(1\;\mathrm{Barrer}=10^{-10}\frac{{\mathrm{cm}}^{3}\mathrm{STP}\cdot \mathrm{cm}}{{\mathrm{cm}}^{2}\cdot s\cdot \mathrm{cmHg}}$)			
%wt.	40 Bar	50 Bar	60 Bar	70 Bar
0	6.5	13.2	7.4	8.5
10	2.4	9.8	12.2	13.0
20	3.8	4.2	2.9	3.4
30	21.2	20.6	22.3	9.0

## Data Availability

All data are included in the article.

## Supplementary Materials

The following supporting information can be downloaded at: https://www.mdpi.com/article/10.3390/polym16040554/s1, Figure S1. Commercial polymeric membranes used in industrial CO_2_ separation processes; Scheme S1. Metathesis reaction between choline chloride ([Ch^+^][Cl^−^]) and sodium hydroxide (NaOH) in ethanol under stirring for 2 h (Stage 1) and neutralization reaction between choline hydroxide ([Ch^+^][OH^−^]) and glycine to form the desirable [Ch^+^][Gly^−^] IL and water as a by-product (Stage 2); Figure S2. Choline chloride and sodium hydroxide in EtOH at t = 0 h and t = 2 h. Note that NaCl precipitated as white powder (Stage 1) and choline hydroxide and glycine in EtOH at t = 0 h and t = 2 h (Stage 2); Figure S3. ^1^H NMR spectra (600 MHz) of the synthesized [Ch^+^][Gly^−^] in D_2_O; Figure S4. ^13^C NMR spectra of the synthesized [Ch^+^][Gly^−^] in D_2_O; Figure S5. Composite membrane film preparation using the solution casting method; Figure S6. Sketch of the experimental setup that was used for the sorption measurements: 1: high-pressure gas tank; 2: cooler; 3: syringe pump; 4: oven; 5: high-pressure cell; Figure S7. CO_2_ desorption from CA doped with 10% wt. [Bmim^+^][HSO_4_^−^] at 25 °C and atmospheric pressure after exposure to a CO_2_ atmosphere at 35 °C and 40 bar (a) and extrapolation to time zero using the FD model (b); Figure S8. Degree of crystallinity calculation of the CA doped with 20% wt. [Bmim^+^][HSO_4_^−^] using the Gaussian function to determine the crystalline peak and the amorphous halo. References [83,84] are cited in the supplementary materials.
