# Supplementary material for: Cellulose Acetate–Ionic Liquid Blends as Potential Polymers for Efficient CO2 Separation Membranes
Source: Polymers (Basel). 2024 Feb 18;16(4):554. doi: 10.3390/polym16040554 (PMC10891773; doi:10.3390/polym16040554)
Supplement: Supplementary file 1 [file polymers-16-00554-s001.zip › polymers-2867808-supplementary.pdf]

polymers

MDPI

Supplementary Information for

Cellulose Acetate-Ionic Liquid Blends as Membranes for Efficient CO_2 Separation

Giannis Kontos[1], Costas Tsioptsias[1,2]* and Ioannis Tsivintzelis[1,*]

[1] Department of Chemical Engineering, Aristotle University of Thessaloniki, 54124 Thessaloniki, Greece
[2] Department of Chemical Engineering, University of Western Macedonia, 50132, Kozani, Greece
* Correspondence: ktsiopts@gmail.com (C.T.), tioannis@cheng.auth.gr (I.T.)

S1. Commercial polymeric membranes used in industrial CO_2 separation processes

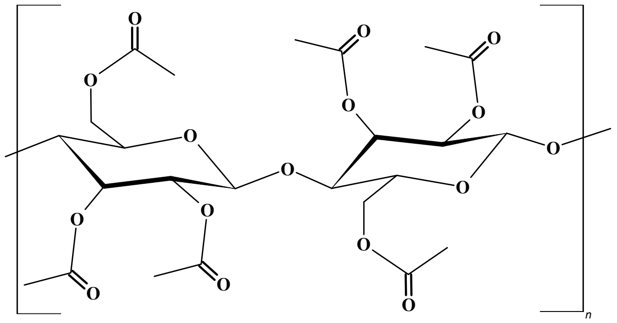

Cellulose triacetate, CTA

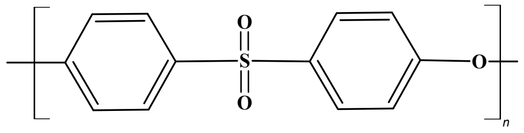

Victrex®, Ultrason® E

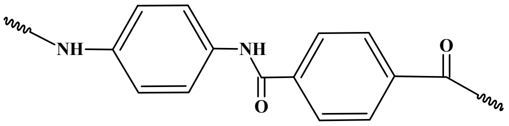

Kevlar®

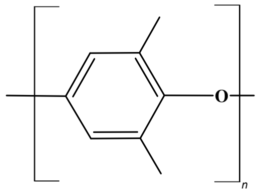

Poly (2,6-dimethyl-1,4-phenylene oxide), PPO

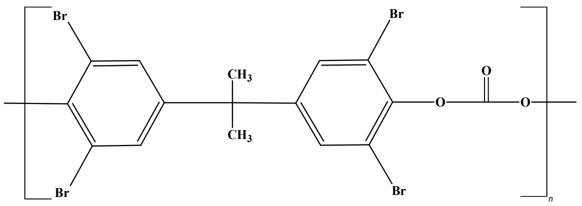

Tetrabromobisphenol A

Figure S1. Commercial polymeric membranes used in industrial CO_2 separation processes.

S2. [Ch][Gly] synthesis

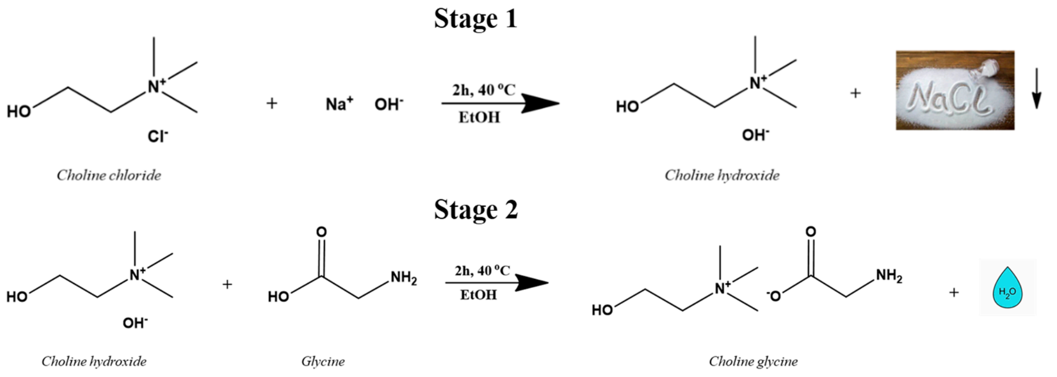

Scheme 1. Metathesis reaction between choline chloride, $[Ch^+][Cl^-]$, and sodium hydroxide, NaOH, in ethanol under stirring for 2 hours (Stage 1) and neutralization reaction between choline hydroxide, $[Ch^+][OH^-]$, and glycine, to form the desirable $[Ch^+][Gly^-]$ IL and water as by-product (Stage 2).

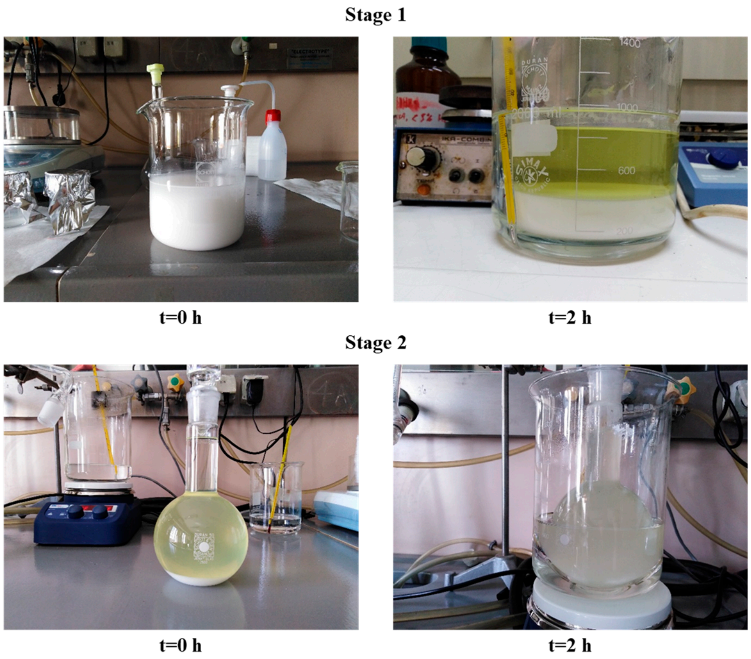

Figure S2. Choline chloride and sodium hydroxide in EtOH at t=0h and t=2h, note that NaCl precipitated as white powder (Stage 1) and choline hydroxide and glycine in EtOH at t=0h and t=2h (Stage 2).

^{1}H and ^{13}C NMR of the synthesized $[Ch^+][Cl^-]$ was carried out at room temperature on a Varian 600 MHz spectrometer in D_2O with tetramethylsilane as internal standard. NMR data is reported as s = singlet, d = doublet, t = triplet, q = quatriplet and m = multiplet or unresolved, while chemical shifts (δ) values are given in ppm. The chemical shifts at 3.49-3.56 ppm (2H, m, CH_2OH), 4.03-4.10 ppm (2H, m, CH_2CH_2N) and 3.21 ppm (9H, s, $(CH_3)_3N$) originate from choline cation, whereas the signal at 3.26 ppm (2H, s, CH_2NH_2) from glycine anion (Figure S3). These results are consistent with literature findings [1-3]. Similar information can also be obtained from ^{13}C NMR analysis. The signals at 56.71 ppm (CH_2OH), 70.33 ppm (CH_2CH_2N) and 58.44 ppm ($(CH_3)_3N$) are attributed to the carbon atoms of the choline cation, while those at 46.80 ppm (CH_2NH_2) and 182.82 ppm (C=O) to

carbons atoms of the glycine anion (Figure S4). These results are, also, in good agreement with the literature [2].

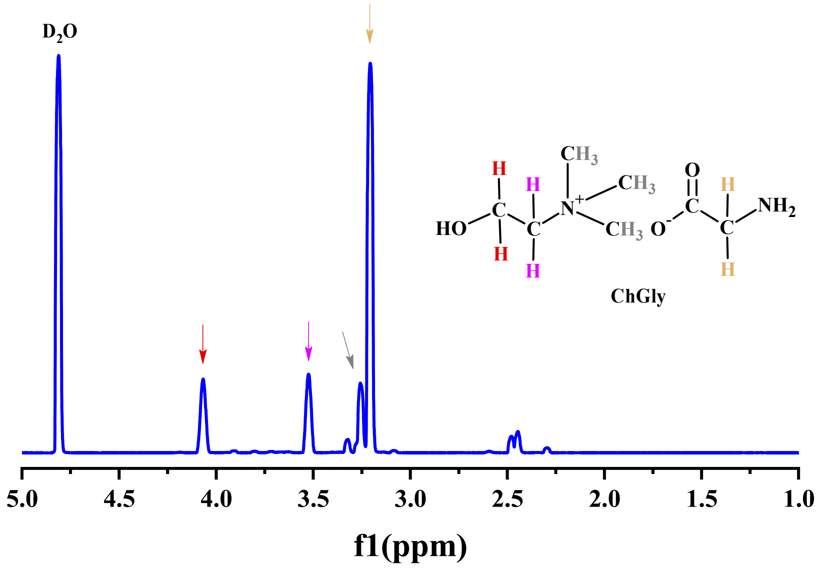

Figure S3. ^1H NMR spectra of the synthesized [Ch^+][Gly^-] in D_2O.

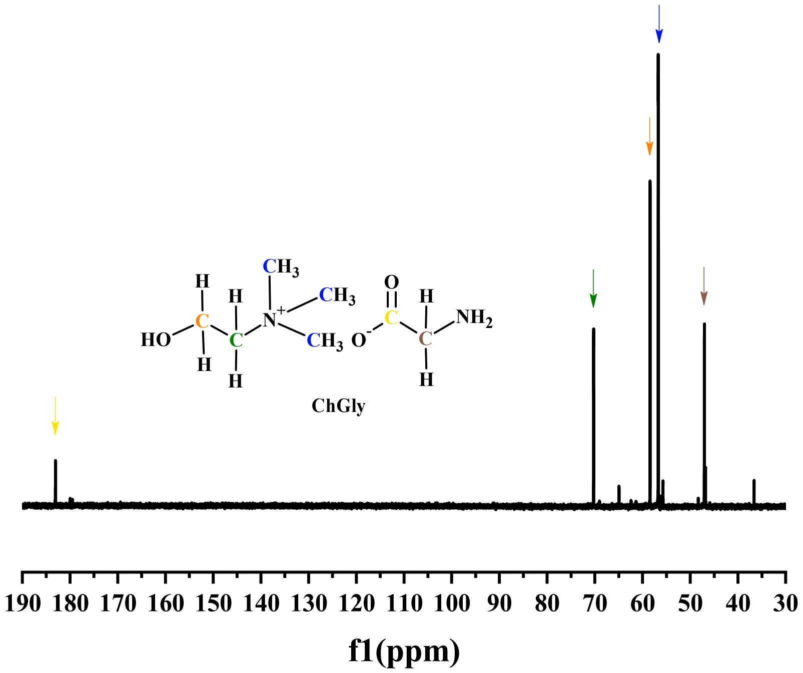

Figure S4. ^{13}C NMR spectra of the synthesized [Ch^+][Gly^-] in D_2O.

S3. CA-IL films preparation

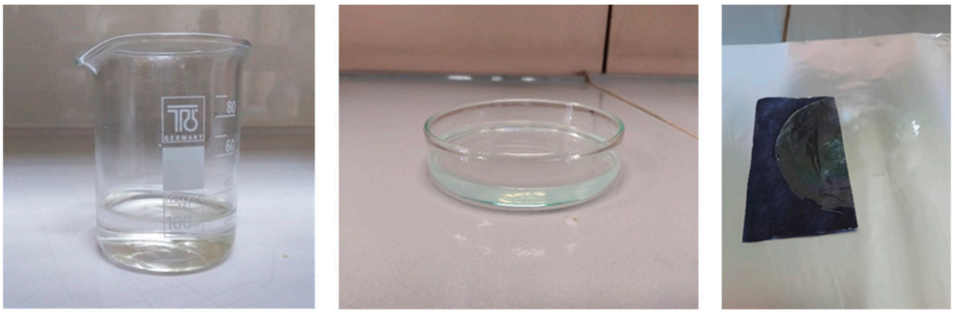

Figure S5. Composite membrane films preparation using the solution casting method.

S4. CO_2 and N_2 sorption measurements using mass loss analysis (MLA)

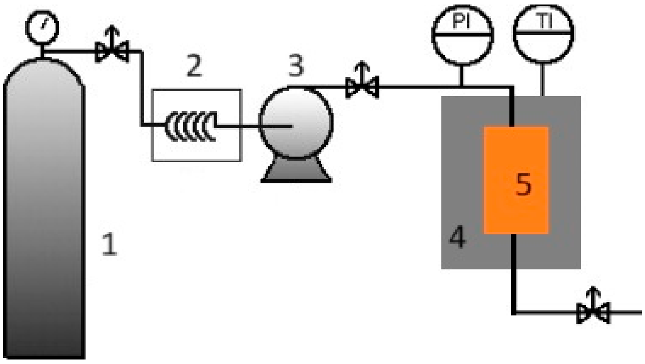

Figure S6. Sketch of the experimental setup that was used for the sorption measurements. 1: High pressure gas tank; 2: cooler; 3: syringe pump; 4: oven; 5: high pressure cell.

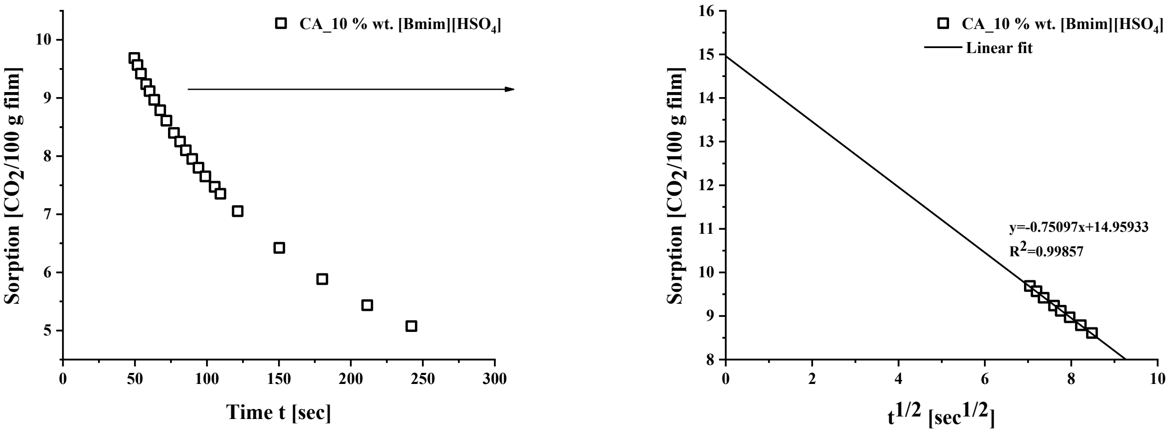

(**a**) (**b**)

Figure S7. CO_2 desorption from CA doped with 10% wt. [Bmim$^+$][HSO$_4^-$] at 25°C and atmospheric pressure after exposure at a CO_2 atmosphere at 35°C and 40 bar (a) and extrapolation to time zero using the FD model (b).

S5. Degree of crystallinity calculation of the CA-IL films using XRD analysis

After subtraction of a linear background, the experimental diffraction curve was resolved into a crystalline peak and an amorphous halo by fitting a theoretical curve. Both the crystalline peak and the amorphous halo were represented by a Gaussian distribution (Figure

S8). The degree of crystallinity was calculated as the ratio of the area of the crystalline peak to the total area [5,6].

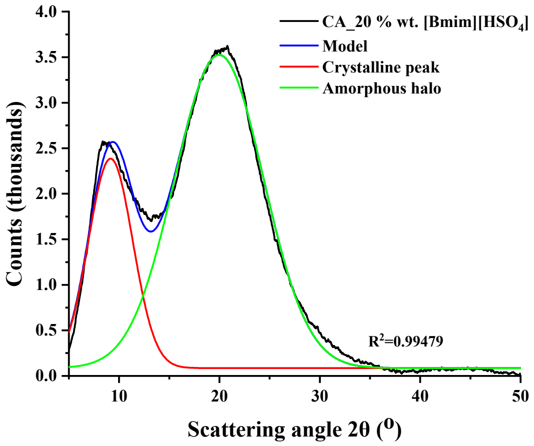

Figure S8. Degree of crystallinity calculation of the CA doped with 20% wt. [$Bmim^+$][HSO_4^-] using Gaussian function to determine the crystalline peak and the amorphous halo.

References

1. Liu, Q.-P.; Hou, X.-D.; Li, N.; Zong, M.-H. Ionic liquids from renewable biomaterials: synthesis, characterization and application in the pretreatment of biomass. *Green Chemistry* **2012**, *14*, 304-307.
2. Moriel, P.; García-Suárez, E.J.; Martínez, M.; García, A.B.; Montes-Morán, M.A.; Calvino-Casilda, V.; Bañares, M.A. Synthesis, characterization, and catalytic activity of ionic liquids based on biosources. *Tetrahedron Letters* **2010**, *51*, 4877-4881.
3. Yuan, S.; Yang, Z.; Ji, X.; Chen, Y.; Sun, Y.; Lu, X. CO2 absorption in mixed aqueous solution of MDEA and cholinium glycinate. *Energy & Fuels* **2017**, *31*, 7325-7333.
4. Felder, R.; Huvard, G. 17. Permeation, diffusion, and sorption of gases and vapors. In *Methods in experimental physics*; Elsevier: 1980; Volume 16, pp. 315-377.
5. Hermans, P.; Weidinger, A. Estimation of crystallinity of some polymers from x-ray intensity measurements. *Journal of Polymer Science* **1949**, *4*, 709-723.
6. Krimm, S.; Tobolsky, A.V. Quantitative x-ray studies of order in amorphous and crystalline polymers. Quantitative x-ray determination of crystallinity in polyethylene. *Journal of Polymer Science* **1951**, *7*, 57-76.
